# A Discrete-Event, Simulated Social Agent-Based Network Transmission (DESSABNeT) model for communicable diseases: Method and validation using SARS-CoV-2 data in three large Australian cities

**DOI:** 10.1371/journal.pone.0251737

**Published:** 2021-05-21

**Authors:** Nicolas J. C. Stapelberg, Nicolas R. Smoll, Marcus Randall, Dinesh Palipana, Bryan Bui, Kristine Macartney, Gulam Khandaker, Andre Wattiaux

**Affiliations:** 1 Gold Coast Health, Southport, Queensland, Australia; 2 Bond University Faculty of Health Sciences & Medicine, Robina, Queensland, Australia; 3 Melbourne School of Population and Global Health, University of Melbourne, Carlton, Victoria, Australia; 4 Central Queensland Public Health Unit, Central Queensland Hospital and Health Service, Rockhampton, Queensland, Australia; 5 Bond University Business School, Robina, Queensland, Australia; 6 National Centre for Immunisation Research and Surveillance (NCIRS), Westmead New South Wales, Australia; Texas A&M University College Station, UNITED STATES

## Abstract

**Importance:**

During pandemics Agent Based Models (ABMs) can model complex, fine-grained behavioural interactions occurring in social networks, that contribute to disease transmission by novel viruses such as SARS-CoV-2.

**Objective:**

We present a new agent-based model (ABM) called the Discrete-Event, Simulated Social Agent based Network Transmission model (DESSABNeT) and demonstrate its ability to model the spread of COVID-19 in large cities like Sydney, Melbourne and Gold Coast. Our aim was to validate the model with its disease dynamics and underlying social network.

**Design:**

DESSABNeT relies on disease transmission within simulated social networks. It employs an epidemiological SEIRD+M (Susceptible, exposed, infected, recovered, died and managed) structure. One hundred simulations were run for each city, with simulated social restrictions closely modelling real restrictions imposed in each location.

**Main outcome(s) and measure(s):**

The mean predicted daily incidence of COVID-19 cases were compared to real case incidence data for each city. *R*_*eff*_ and health service utilisation outputs were compared to the literature, or for the Gold Coast with daily incidence of hospitalisation.

**Results:**

DESSABNeT modelled multiple physical distancing restrictions and predicted epidemiological outcomes of Sydney, Melbourne and the Gold Coast, validating this model for future simulation work.

**Conclusions and relevance:**

DESSABNeT is a valid platform to model the spread of COVID-19 in large cities in Australia and potentially internationally. The platform is suitable to model different combinations of social restrictions, or to model contact tracing, predict, and plan for, the impact on hospital and ICU admissions, and deaths; and also the rollout of COVID-19 vaccines and optimal social restrictions during vaccination.

## Introduction

Agent-based modelling (ABM) is an approach to simulating complex systems including the COVID-19 pandemic [1]. ABMs consist of a simulated system of agents (people), the network of relationships (contact) between them, and where they simulate disease transmission, the disease dynamics that inform disease spread.

They can thus model social networks and complex behavioural interactions in a granular fashion, as well as stochastic processes which shape disease transmission in pandemics such as COVID-19 [2]. This work focuses on describing a Discrete-Event, Simulated Social Agent-Based Network Transmission (DESSABNeT) platform. DESSABNeT builds life-like social networks for a population of agents based on demographic data and models the transmission of a communicable disease throughout a network of agents producing simulated epidemiological and medical outcomes and an effective reproductive number (R_eff_).

Underlying the model is a SEIRD+M (Susceptible, exposed, infected, recovered, died and managed) structure, with heterogeneous mixing that can be affected by non-pharmacological interventions (NPIs). Interactions between the societal structure, the NPI’s and disease dynamics result in 1) Epidemiological curves for the SEIRD+M compartments; 2) *R*_*eff*_ for the simulation and 3) Outputs such as hospitalisations, intensive care unit (ICU) admissions and deaths. We demonstrate simulated transmission of COVID-19 in three outbreaks in Australian cities: Melbourne and Sydney, which saw substantial outbreaks and Gold Coast, which saw a more limited COVID-19 outbreak. We also demonstrate the effect of non-pharmacological interventions on the outputs of this model.

## Methods

This work was assessed by the Gold Coast Hospital and Health Service Human Research Ethics Committee and received a formal waiver for full ethical review (LNR/2020/QGC/65664).

### Agents

DESSABNeT, programmed in MATLAB [3], models disease spread through social networks. ABMs simulate artificial societies composed of “agents” which in this context represent cyber individuals with particular demographic characteristics and an initial disease state [4]. DESSABNeT agents 1) belong to predetermined social networks where each agent is linked to other agents in family relationships, friendship and kin relationships and work or education relationships and 2) have predetermined but changeable (for different restrictions) weekly schedules that determine not only the frequency of family, social and workplace contacts, but also separate random contacts in large, medium and small contact groups. The ability to change these schedules multiple times during a simulated outbreak allows a wide range of social restrictions to be modelled. The number of agents was set to the population size of each city, with no births or competing causes of death (non-COVID-19 deaths) modelled.

### Containers

Each individual agent in a population is given a unique identity based on actual demographic data for a given city (e.g. Sydney, Melbourne, Gold Coast). Agents become nodes in a social network including aforementioned key family, friendship and kin, and primary activity sub-networks (the latter comprising pre-school, school or vocational education, work or retirement) based on discrete random probabilities informed by census data [5–9]. These sub-networks are termed Containers (*C*_*x*_; see Table 1) and in total represent family units (*C*_*1*_), friend/social network units (*C*_*4*_), primary activity units (*C*_*2*_), public transport units (*C*_*3*_), essential activities units (food shopping, medical visits) (*C*_*5*_), medium leisure gathering units (*C*_*6*_), and large public gathering units (*C*_*7*_). Each container has a unique identification code which is assigned to each agent within that container. Containers have varying sizes, some allowing random interaction for public leisure activities and small, medium and large group gatherings.

**Table 1:** Containers used to build the social network.

	Container	Examples	Container Size	Age Group	Transmission Probability
**C_1_**	Family		Unique family groups (N = 1–6)	0 to 19	0.0748
				20 and older	0.0748
**C_2_**	Classroom/educational and work	Daycare, kindergarten, school classrooms, working teams, office staff	Educational groups (M = 25, SD = 2), Work groups (M = 10, SD = 4)	0 to 4	0.00391
				5 to 19	0.00391
				20 and older	0.034
**C_3_**	Public transport	Bus, train or tram, ride-share service, etc.	Small Exposure Groups, N = 20 Fixed	0 to 4	0.0121975
				5 to 19	0.0121975
				20 and older	0.0121975
**C_4_**	Social contacts (friends, kin)	Social visits by friends or kin (extended family)	Agents belonging to unique social groups (M = 30, SD 2)	0 to 4	0.068
			Visits M = 3, SD = 1	5 to 19	0.068
				20 to 64	0.068
				64 and older	0.068
**C_5_**	Essential activities	Medical and pharmacy visits, food shopping, other essential activities	Medium Exposure Groups, N = 100 Fixed	0 to 4	0.009758
				5 to 19	0.009758
				20 to 64	0.009758
				64 and older	0.009758
**C_6_**	Medium-sized group leisure activities	Eating out, playing sport or going to the cinema	Medium Exposure Groups, N = 100 Fixed	0 to 4	0.009758
				5 to 19	0.009758
				20 to 64	0.009758
				64 and older	0.009758
**C_7_**	Large-group leisure activities	Visiting professional sporting events, large exhibitions, attending large clubs, amusement parks or shopping centres	Large Exposure Groups, N = 500 Fixed	0 to 4	0.0004879
				5 to 19	0.0004879
				20 to 64	0.0004879
				64 and older	0.0004879

(Transmission probability is expressed in *C*_*1*_ as the *infective agent* being aged 0–19 and 20 and over. All other transmission probabilities are expressed in terms of the *receiving agent* being exposed to transmission, as per Chao et al. [10].

Agents cannot interact with agents outside their current container. Each container has a specific transmission probability (Table 1).

Family containers (*C*_*1*_) were created using the discrete probability distribution based on household sizes for each city of interest [5–7]. The friend and kin container (*C*_*4*_) was populated with the understanding that 97% of persons have approximately 25 or fewer very strong social ties or 40 somewhat strong social ties [11]. We chose a total of 30 agents for these containers (suggestive of 10 kin, 15 friends and 5 friends from work). Work containers (*C*_*2*_) were populated using a discrete probability distribution of workplace and classroom sizes based on the literature [12,13].

In their work time agents either attend a primary activity (work or education) container whereas retired agents pursue leisure activities or remain at home. While work/education containers may represent an entire workplace or educational campus, the size of the workplace container only represents the small proportion of those agents coming into regular contact with each other (as defined for contact tracing) [14].

Agents may have several daily encounters in groups of different sizes. The public transport container (*C*_*3*_) is populated with up to 20 agents, recognizing that similar agents are likely to use the same transport on the way to and from work. However, absolute numbers of public transport users are determined by demographic data [5–7]. Essential activities containers (*C*_*5*_) represent shopping for food and other essentials, pharmacy and medical visits with potential contact with up to 100 agents. Medium groups (*C*_*6*_) are containers with up to 100 agents and represent people gathering in large stores, pubs or restaurants. Large public gathering containers (*C*_*7*_) represent substantial gatherings during sports and entertainment events, or large shopping venues, with up to 500 agents. We restricted the size of *C*_*7*_ to 500 as the outbreak events we modelled had a restriction on gatherings of over 500 people as a very early measure.

### Weekly schedules

DESSABNeT creates itineraries based on three periods of activity per day loosely corresponding to time-periods of morning, afternoon and evening. For this simulation, the entering of a container is more important than the time spent in the container, as each activity is associated with a transmission probability determined by the container in which that activity occurs, rather than the duration of contact. Itineraries for each agent were established for a 7-day week, with work, leisure and social activities cumulatively corresponding to demographic data and the literature. Each agent’s weekly schedule is repeated for the duration of the simulation, however changes in government-imposed restrictions can alter agent schedules, affecting disease transmissibility or *R*_*eff*_.

The morning period (*work time*), was allocated to children attending school, pre-school or day-care, and most adults going to work. To simulate shift work, agents could work during the “morning” or “afternoon” period. Adults already working from home pre-pandemic were modelled [15]. Afternoons (*discretionary time*) were allocated for essential activities (*C*_*5*_), family time (*C*_*1*_), social visits (*C*_*4*_), medium (*C*_*6*_) and large (*C*_*7*_) group activities according to statistics for daily activities [16,17]. Evenings and nights (*social time*) were allocated to mixing in family or social containers (*C*_*1*_, *C*_*4*_) but typically only allowing on average 3 people from the social network of approximately 30 friendship and kin to visit at any one time, with 1 to 5 social visits per week [11,18]. Further complexities were introduced in the model: Agents using public transport (determined by public transport statistics for each city) travelled on the days that they worked (the public transport container being implemented during discretionary time) [5–7,19]. If children were not attending school, vocational or educational institutes (e.g. on weekends), they were allocated to the same (non-work) activity as one of their carers/parents, thus simulating children accompanying an adult on an outing.

### Modelling physical distancing

Modelling physical distancing was implemented by either altering agent schedules (e.g. restricting access to work containers when working from home) or introducing a factor to modify transmission probability [20], which was used to simulate wearing masks in Melbourne. Schedule changes such as agents working from home reduces the number of people in workplace and public transport containers. Modifying discretionary time allocations can also simulate restrictive measures, e.g. reducing the percentage of time spent at medium or large group activities. DESSABNeT can model school closures or alter the number of friend and social visits. The number of essential activities per week was kept constant throughout social restrictions, as reflected in Google mobility data [21].

### DESSABNeT compartment model (SEIRD+M)

**Fig 1:**
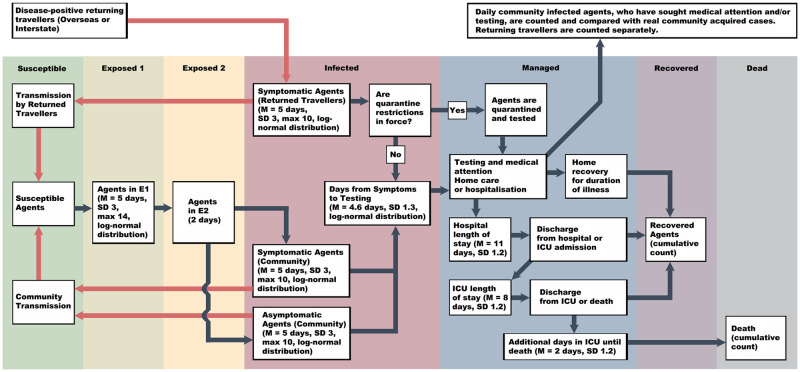
The flow of agents through the different SEIRD+M compartments. All agents are assumed to be susceptible at the start, and quarantine restrictions come into force based on when formal quarantine was announced in the city being modelled.

DESSABNeT employs a S, E_1_, E_2_, I_a_, I_s_, R, D, M (Susceptible, exposed (latent un-infectious and latent infectious), infected (asymptomatic and symptomatic), recovered, died and managed) structure (Fig 1). We modelled a closed system with no births or general mortality. Modelling social restrictions (i.e. working from home) changes the contact rates between agents, thus changing the transmission rate over time, hence: *f*(*β*_*t*_). The following ordinary differential equation would closely represent our ABM, except that various particulars are simplified here (e.g. the managed compartment is split into numbers of persons isolating at home, in hospital or ICU).

$$\frac{dS}{dt}=-\frac{f(\beta_{t})IS}{N}$$

$$\frac{dE_{1}}{dt}=\frac{f(\beta_{t})IS}{N}-a_{1}E_{1}-\delta_{1}E_{1}$$

$$\frac{dE_{2}}{dt}=a_{1}E_{1}-a_{2}E_{2}-\delta_{2}E_{2}$$

$$\frac{dI_{a}}{dt}={(1-k)a}_{2}E_{2}-\gamma_{1a}I_{a}$$

$$\frac{dI_{s}}{dt}=ka_{2}E_{2}-\gamma_{1s}I_{s}-\mu_{1s}I_{s}-\delta_{3}I_{s}$$

$$\frac{dR}{dt}=\gamma_{1a}I_{a}+\gamma_{1s}I_{s}+\gamma_{2}M$$

$$\frac{dD}{dt}=\mu_{1s}I_{s}+\mu_{2}M$$

$$\frac{dM}{dt}=\delta_{1}E_{1}+\delta_{2}E_{2}+\delta_{3}I_{s}-\gamma_{2}M-\mu_{2}M$$

- *a*_1_*E*_1_ represents the transfer from the latent non-infectious phase to the latent infectious (pre-symptomatic infectious) compartment.
- *a*_2_*E*_2_ represents the transfer rate from the exposed (latent and latent infectious) compartments to the infective compartment.
- *I*_*a*_, *I*_*s*_ are the asymptomatic and symptomatic infected containers. We follow Buitrago-Garcia [22] in modelling 15% of agents as asymptomatic, which impacts on their transmission rate (not parametrized here for simplicity). Asymptomatic infected agents (*I*_*a*_) do not enter the managed compartment and do not die.
- *δ*_1_*E*_1_, *δ*_2_*E*_2_, *δ*_3_*E*_*s*_ represents the transfer rate from the *E*_1_, *E*_2_, or *I*_*s*_ compartments respectively into the managed compartment. Typically, those leaving the *E* compartments are due to the efforts of contact tracing.
- *γ*_1_, *γ*_2_ represents the transfer rates from the infection compartment to the recovered compartment.
- *μ*_1_, *μ*_2_ represents the transfer rates from the asymptomatic infected (*μ*_1*a*_), symptomatic infected (*μ*_1*s*_) and managed (*μ*_2_) compartments to the dead compartment.

The parameters *α*, *γ* and *μ* could be replaced by the parameters of a different distribution such as Erhlang or log-normal distribution, which may better represent the distributions seen in the transfer between compartments.

### Transmission probabilities

As a key point of difference with differential equation-based compartment models, this platform estimates a society and disease-specific net transmission rate through simulation, rather than using an *a priori* assumption. Heterogeneous mixing occurs in a variety of container types, with specific transmission probabilities (TP)s for each infected-to-susceptible agent encounter (see Table 1).

TPs govern the transfer of agents from S to E1. TPs represent the probability that one agent in *E*_2_ or *I* will transmit the disease to a susceptible agent. Other COVID-19 ABM’s have used TPs from the influenza literature [1]. We aimed to generate TPs that were model and COVID-19 specific, based on an overall basic reproductive number of 2.5. We divided 2.5 by the number of containers entered by one infected agent during the mean infective period (*E*_2_ + *I*; 5.807 days), multiplied by 3 containers per day, which is 17.4 containers per infective period, or 0.143 transmissions per container. The transmission probability for a container type, was 0.143 divided by the average number of agents in that container. In some contexts, we slightly modified TPs to ensure that transmission scenarios resembled real life situations. For example, school transmissions were derived from the literature (0.0023), and medium group container TP was multiplied by a factor of 4 to better represent closer social contact (e.g. café, bars or dining scenarios with friends) [23]. Finally, a scaling factor of 1.7 was applied to all TPs (similarly done by Chang et al. and others [1], resulting in the final values seen in Table 1.

We show the flow of agents through the SEIRD+M compartments in Fig 1 and the disease dynamics that govern the transfer of agents between compartments, or within the managed compartment, in Table 2. The managed compartment models quarantine (as imposed by regulations for returning travellers or contact tracers), self-isolation for symptomatic persons recovering at home, and hospitalisation. The duration of transmission, or infectivity period, is the duration of *E*_2_ + *I* −*M*.

**Table 2:** Disease dynamics parametrisation of disease course and characteristics of COVID-19.

Description	Value
Percentage asymptomatic cases in the population [1,22]	15%
Percentage asymptomatic transmission relative to symptomatic transmission [1]	30%
Exposed duration (E1 + E2) (days, log-normal distribution) [24–26]	5 (SD 3), max 14
Exposed duration 2 (E2) (days) [27]	2
Infectivity duration (I) (days) [28]	5 (SD 3), max 10
Delay from symptom onset to medical assessment and COVID-19 testing (days, log-normal distribution) [24]	4.6 (SD 1.3)
Average length of stay in medical ward beds (days) [28]	11
Average length of stay in ICU (days) [28]	8
Average additional length of ICU stay with subsequent inpatient mortality (days) [28,29]	2
Mean percentage of symptomatic infected requiring hospitalisation for age 0–4 (SD = 1.2, log-normal distribution) [28,29]	0.06
Mean percentage of symptomatic infected requiring hospitalisation for age 5–19 (SD = 1.2, log-normal distribution) [28,29]	0.06
Mean percentage of symptomatic infected requiring hospitalisation for age 20–64 (SD = 1.2, log-normal distribution) [28,29]	5.874
Mean percentage of symptomatic infected requiring hospitalisation for age over 65 (SD = 1.2, log-normal distribution) [28,29]	44.63
Mean percentage of admitted cases requiring ICU for age 0–4 (SD = 1.2, log-normal distribution) [28,29]	33.333
Mean percentage of admitted cases requiring ICU for age 5–19 (SD = 1.2, log-normal distribution) [28,29]	33.333
Mean percentage of admitted cases requiring ICU for age 20–64 (SD = 1.2, log-normal distribution) [28,29]	29.385
Mean percentage of admitted cases requiring ICU for age over 65 (SD = 1.2, log-normal distribution) [28,29]	29.363
Mean percentage mortality in those admitted to ICU for age 0–4 (SD = 1.2, log-normal distribution) [28,29]	0.001
Mean percentage mortality in those admitted to ICU for age 5–19 (SD = 1.2, log-normal distribution) [28,29]	0.1
Mean percentage mortality in those admitted to ICU for age 20–64 (SD = 1.2, log-normal distribution) [28,29]	3.544
Mean percentage mortality in those admitted to ICU for age over 65 (SD = 1.2, log-normal distribution) [28,29]	31.9

For the purposes of our simulation we used a 15% asymptomatic infection rate and the relative infectiousness of asymptomatic carriers was 30% compared to symptomatic cases [22].

Agents in the managed container of the model do not transmit disease. The time from first symptom to diagnosis represents the time from the start of the agent’s time in the infectious (*I*) compartment to the time of entering the managed compartment (*M*). Within the managed compartment agents were allocated to treatment at home, hospitalised status, ICU admission and death based on age-based probabilities derived from the literature and detailed in Table 2.

The transmission rate (contact frequency multiplied by the transmission probability) is an emergent output of the simulation, dependent on input demographic and societal parameters (e.g. number of persons per household or workplace sizes), stochastic social network interactions (e.g. attending large gatherings) and disease dynamics.

### Basic/effective reproduction numbers and transmission rates

The basic reproduction number and effective reproductive number in the presence of non-pharmacological interventions is calculated using a Who Acquires Infection from Whom or WAIFW matrix. The dominant eigenvalue of the matrix represents the basic or effective reproduction number for the simulated system (e.g. Sydney without restrictions).

The overall next generation matrix for a population setup in this system begins with a social contact matrix (*H*). Each container (*C*_1−7_) has unique contact patterns represented by *H*_*ij*_. Matrix *H*_*ij*_ represents the number of daily contacts a person of age category *i* has with persons of age category *j*. The transmission rate (*β*_*ij*_) also known as age category specific *R*_*o*_, is the contact rate (*H*_*ij*_) multiplied by the TP of the infectee resulting in a matrix (*G*_*ij*_):

$$G_{ij}=\left[\begin{array}{ccc} \beta_{11} & \beta_{12} & \beta_{13}\\ \beta_{21} & \beta_{22} & \beta_{23}\\ \beta_{31} & \beta_{32} & \beta_{33} \end{array}\right]$$

In this ABM, each agent will spend time in three different containers each day. The resultant transmission rate matrix per day is the sum of the transmission rates for each active container (containers that people enter), divided by the total number of active containers at each of the three time periods. For example, matrix *G*_*ij*_ uses 3 age categories (3 types of infectors and infectees), and *β*_12_ represents the number of infections a single agent of age category 1 causes on average in persons of age category 2, e.g. a child infecting an adult, and averaged over the course of a week.

The basic reproduction number is the dominant eigenvalue of the WAIFW matrix *G*_*ij*_ multiplied by the infectivity period of the system. The effective reproduction number is calculated similarly, except that NPI’s will change the mixing of agents (e.g. working from home), and the infectivity period. The duration of the infectivity period is altered as *E*_2_ + *I* is reduced when persons enter the managed compartment as a result of contact tracing, self-isolation and hospitalization, where no transmission occurs.

The *emergent* transmission rate is calculated daily. The emergent transmission rate is the total number of transmissions per day, divided by the number of agents who could transmit the illness minus agents in the managed compartment where transmissions cannot occur (total of agents in: *E*_2_ + *I*_*s*_ + *I*_*a*_ − *M*). The daily transmission rate multiplied by the infectivity period (duration of *E*_2_ until person enters the managed compartment) also represents the *emergent* reproduction number (*R*_*emerg*_):

$$R_{emerg}=\frac{Total\;Transmissions}{\left(E_{2}+I_{s}+I_{a}-M\right)}\times duration\;of\;infectiousness$$

In the ABM, a person can be in the *I*_*s*_ compartment and be self-isolating in the managed *M* compartment.

### Outputs

The model produces daily prevalence and incidence values for each of the SEIRD+M compartments. For the managed compartment, daily incidence and prevalent COVID-19-positive agents treated at home, hospitalisations, ICU admissions and deaths are recorded, as well as daily prevalence and incidence of agents quarantined. Daily overall, and container specific, incidence of transmissions and cumulative transmissions are recorded. Pandemic outbreak was simulated in each city 100 times and the median values for these simulations were used to present outputs.

Medical outcomes were calculated using estimates of the fraction of symptomatic infected hospitalised, hospitalised people admitted to ICU and deaths as a fraction of those admitted to ICU, derived from Moss at al. and Zhou et al. [28,29] and presented in Table 2.

Symptomatic infected agents enter the medical portion of the Managed container after a specified number of days to seeking medical attention as quantified by Li et al. [24], calculated as a log normal distribution for each agent. The percentage of symptomatic infected admitted is based on age, and is calculated based a log normal probability distribution with a mean of 0.060 (age 0–19), 5.874 (age 20–64) and 44.630 (aged 65 and over), derived from [28,29]. Those not admitted have home-based care but remain in the managed container. Agents admitted to ICU are selected from hospital admissions with based a log normal probability distribution with means per age group shown in Table 2. Deaths are selected from ICU admissions, with probabilities shown in Table 2. The probability of mortality can be expressed as:

$$\text{Pr}\left(\text{Death}\right)=\text{Pr}\left(\text{Death}\left|\text{ICU}\;\text{admission}\right|\text{Hospitalization}\right)$$

Average lengths of stay in medical beds (11 days) [28], and ICU beds (8 days) [28], and additional length of ICU stay with agents who die (2 days) [28,29], were taken from the literature (Table 2).

### Real world simulation and comparisons

We compared predicted outputs from simulation to real daily incidence cases for Sydney, Melbourne and Gold Coast, to demonstrate a discrete outbreak, a ‘multi-wave’ outbreak, and a very limited or controlled outbreak without sustained community transmission. Tests of distributional assumptions (Kolmogorov-Smirnov Tests) were used to assess goodness-of-fit in the presence of non-linear relationships. P-values greater than 0.10 were used to indicate observed vs simulated estimates drawn from similar distributions. We then estimated hospitalizations, ICU bed requirements and deaths (per 100,000 population).

The Sydney outbreak (March 2020) disease dynamic parameters were used to calibrate the DESSABNeT COVID-19 model, with validation on Melbourne and Gold Coast data (see Table 3). The naïve system (no restrictions) had a next generation R_0_ value of 1.45. This decreased to 0.88 in stage 4 restrictions. The settings for each phase result in differing R_0_ derived using the next generation operator method.

**Table 3:** Social restrictions for the Sydney simulation.

Variable	Phase 1 (Normal Social Network)	Phase 2 (SR 1)	Phase 3 (SR 2)	Phase 4 (SR 3)
Day Number	1 to 16	17 to 23	24 to 30	31 to 80
Dates	1 March—16 March, 2020	17 March—23 March, 2020	24 March—30 March, 2020	31 March to 19 May, 2020
Sydney Restrictions	Nil	Public events > 500 people cancelled Overseas travellers must self-isolate 14 days	Pubs, hotels, clubs, restaurants and recreation facilities closed.	People must not leave their residence except for essential purposes.
Restrictions in DESSABNeT	Nil*	All returning travellers placed in 14 day quarantine	↑ HLT, ↑ WFH, ↓ MGA, ↓ LGA, ↓ PTU, ↓ FKV	↑↑ HLT, ↑↑ WFH, 0% MGA, 0% LGA, ↓↓ PTU, ↓↓ FKV
Essential visits per week (N)	3	3	3	3
% large-group activity in leisure time	11.4	11.4	0	0
% medium-group leisure activity in leisure time	40.4	40.4	23	0
% solitary home activity in leisure time	48.2	48.2	77	100
% of agents working from home	30.2	30.2	47.1	64
% of agents using public transport	32.9	32.9	14.1	8
Friend and Kin Contacts per Week (N)	5	5	3	2
School attendance	Schools Open	Schools Open	Schools Open	Schools Open
Median Phase R_0_ (95% CI)	1.447 (0.208, 6.546)	1.447 (0.208, 6.546)	1.104 (0.142, 5.885)	0.881 (0.088, 5.480)

*Maximum large group exposure set at 500 agents from beginning of simulation.

HLT = Home Leisure Time, WFH = Work From Home, MGA = Medium Group Activity, LGA = Large Group Activity, PTU = Public Transport Use, FKV = Friend and Kin Visits.

Changing social network parameters are shown with different restrictions being introduced or lifted. Median phase R_0_ using the next generation operator represents the median basic reproduction number for the contact network with or without social restrictions in place. The 95% CI uses the 2.5^th^ and 97.5^th^ percentile of contact matrix values to provide a range of next generation operator R_0_ values.

Melbourne was simulated from March 2020 to September 2020, across 8 simulated social restriction phases (Table 4), including the relaxation of restrictions in early May and their re-introduction in June and July 2020. The naïve system (no restrictions) had a median next generation R_0_ value of 1.41 (see Table 4). This decreased to 0.75 during the most aggressive restrictions. One challenge was how to simulate the mixing of people in quarantine with members of the community, that occurred around mid-May (and were the subsequent subject of an inquiry) [30]. This was achieved by not placing agents who arrived from overseas in quarantine between day 85 and 104 (24 May, 2020–12 June, 2020, 3 weeks) of the simulation, representing the reported interaction of returned travellers with the community around this time. A further challenge was simulation of the effects of contact tracing in reducing time to enter the managed container and simulating contact tracing capability being placed under pressure. We reduced the infective period by 27% when contact tracing during all simulation phases, except during Melbourne’s second wave, assuming a reduced capacity to contact trace during this time [31,32].

**Table 4:** Social restrictions for the simulation of two Melbourne outbreaks.

Variable	Phase 1 (Normal Social Network)	Phase 2 (SR 1)	Phase 3 (SR 2)	Phase 4 (First Lifting of SR)	Phase 5 (Mixed)	Phase 6 (SR 4)	Phase 7 (SR 5)	Phase 8 (SR 6)
Day Number	1 to 19	20 to 24	25 to 72	73 to 86	87 to 130	131 to 144	145 to 153	154 to 195
Dates	1 March—19 March, 2020	20 March—24 March, 2020	24 March—11 May, 2020	12 May to 25 May, 2020	26 May to 8 July, 2020	9 July to 22 July, 2020	23 July to 31 July, 2020	1 August to 11 September, 2020
Melbourne Restrictions	Nil	Public events > 500 people cancelled Overseas travellers must self-isolate 14 days	Only four reasons to be out: essential activities, care and caregiving, exercise, and study or work. Schools closed 24 March, 2020	First lifting of restrictions: Visiting friends and family allowed with a maximum gathering of up to 10 outdoors and 5 indoors.	Lifting of Restrictions 2: Publicly accessible outdoor communal activity. However, a small number of Melbourne postcodes return to Stage 3 Stay at Home restrictions 2 July to 21 July, 2020 (Day 124 to 130).	Metropolitan Melbourne returns to Stage 3 Stay at Home restrictions.	Metropolitan Melbourne or Mitchell Shire residents must wear a face covering outside of the home.	Only four reasons to be out: essential activities, care and caregiving, exercise, and study or work. Many businesses are required to close.
Restrictions in DESSABNeT	Nil*	All returning travellers placed in 14-day quarantine	↑↑ HLT, ↑↑ WFH, 0% MGA, 0% LGA, ↓↓ PTU, ↓↓ FKV	↑↑ HLT, ↑↑ WFH, 0% MGA, 0% LGA, ↓↓ PTU, ↑ FKV	↓ HLT, ↓ WFH, ↑ MGA, ↑ LGA, ↑ PTU, ↑ FKV	↑ HLT, ↑ WFH, ↓ MGA, ↓ LGA, ↓ PTU, →FKV	Change to beta values**	↑↑ HLT, ↑↑ WFH, 0% MGA, 0% LGA, ↓↓ PTU, ↓↓ FKV
Essential visits per week (N)	3	3	3	3	3	3	3	3
% large-group activity in leisure time	11.4	11.4	0	0	10	10	10	0
% medium-group leisure activity in leisure time	40.4	40.4	0	0	39	27	27	0
% solitary home activity in leisure time	48.2	48.2	100	100	51	63	63	100
% of agents working from home	30.2	30.2	64	64	35	41	41	64
% of agents using public transport	37	37	18.5	18.5	30	25	25	18.5
Friend and Kin Contacts per Week (N)	5	5	2	4	5	5	5	2
School attendance	Schools Open	Schools Open	Schools Closed	Schools Closed	Schools open between Day 100 and 113.	Schools Closed	Schools Closed	Schools Closed
Median Phase R_0_ (95% CI)	1.408 (0.204, 6.394)	1.408 (0.204, 6.394)	0.775 (0.078, 5.075)	0.825 (0.091, 5.162)	1.330 (0.198, 6.118)	1.144 (0.153, 5.894)	1.106 (0.147, 5.809)	0.775 (0.078, 5.075)

*Maximum large group exposure set at 500 agents from beginning of simulation.

** Beta values in each container, except Family containers multiplied by a “physical distancing/ mask wearing factor” of 0.95.

HLT = Home Leisure Time, WFH = Work From Home, MGA = Medium Group Activity, LGA = Large Group Activity, PTU = Public Transport Use, FKV = Friend and Kin Visits.

Changing social network parameters are shown as different restrictions are introduced or lifted. Median phase R_0_ using the next generation operator represents the median basic reproduction number for the contact network, with or without social restrictions in place, for both outbreaks. The 95% CI uses the 2.5^th^ and 97.5^th^ percentile of contact matrix values to provide a range of next generation operator R_0_ values.

We also note the use of modified transmission probabilities from day 145 (23 July, 2020) onwards in the simulation to represent the directive to wear masks. We did not explicitly model the restrictions introduced across only 10 postcodes on 2 July, but adopted global restriction values based on Google Mobility data for the whole of Melbourne.[21]

Similarly, Table 4 demonstrates the setup parameters for the various Gold Coast phases. The naïve system (no restrictions) had a median next generation R_0_ value of 1.41. This decreased to 0.75 during the most aggressive restrictions. Due to the opportunity to access good quality admissions data for the Gold Coast, we were also able to compare estimates of hospitalizations and ICU admissions.

## Results

### Simulations of Sydney, Melbourne and the Gold Coast

**Fig 2:**
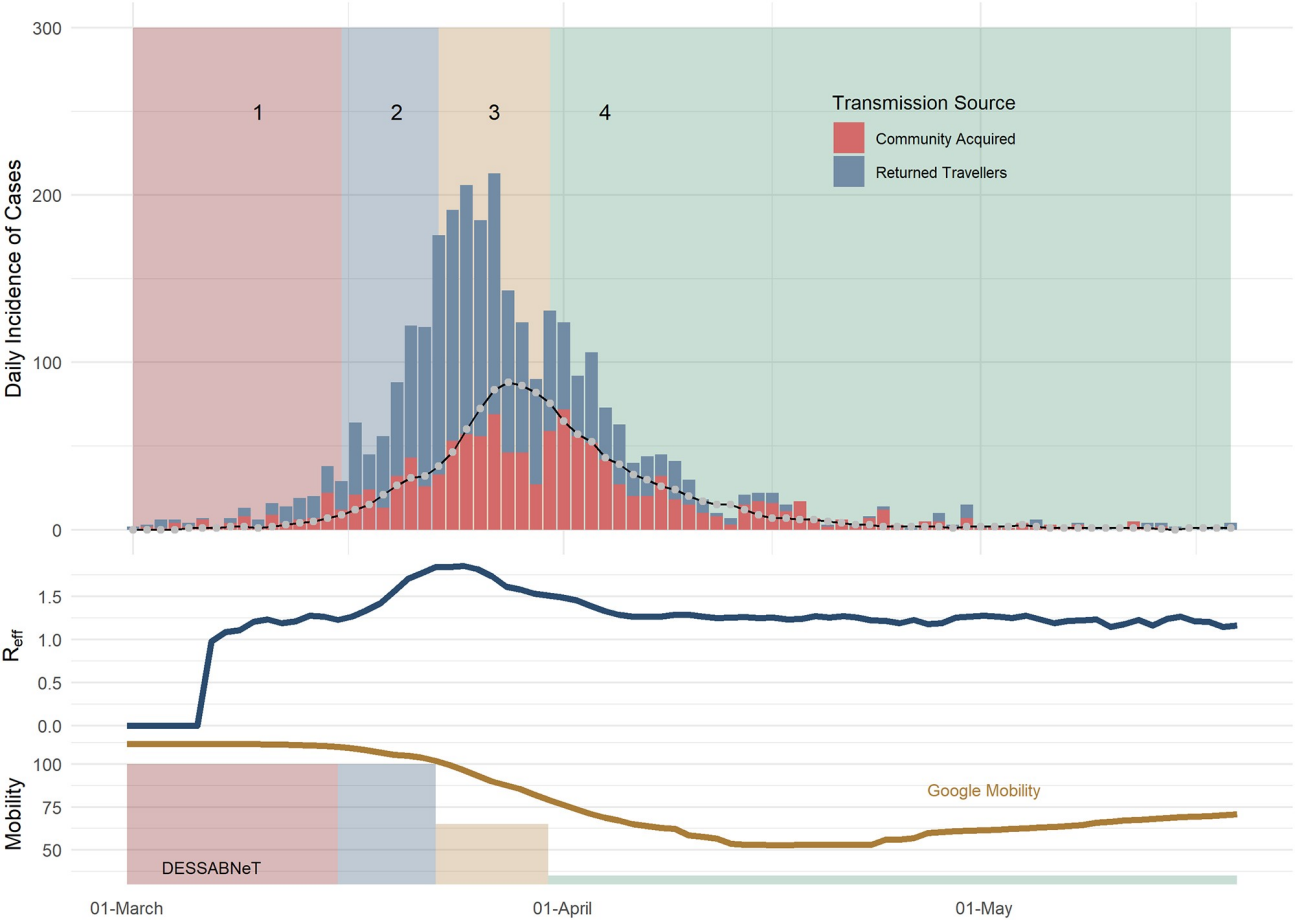
Sydney daily incidence of COVID-19 cases (bars) and simulated daily incidence of symptomatic community transmission cases (line), with the simulated daily *R*_*eff*_ shown in the middle plot. The line in the top plot is the simulated total incidence. The rates of working from home used in our simulation corresponds to Google mobility (workplace) measures.

The Sydney outbreak with a population of 5,312,000 agents was simulated for 80 days (1 March to 19 April), with data obtained from publicly available sources (Fig 2) [33]. Table 3 shows the 4 different restrictions simulated. The observed vs simulated estimates were drawn from the same distribution (p = 0.44). The median, 75^th^ and 95^th^ percentile of the maximum *R*_*eff*_ for each of the 100 simulations was 1.87, 1,94 and 2. Similarly, the median, 75^th^ and 95^th^ percentile of the maximum daily incidence for the 100 simulations was 123, 133 and 143, respectively, which is above what was seen in the Sydney outbreak (maximum daily community transmissions was 72).

**Fig 3:**
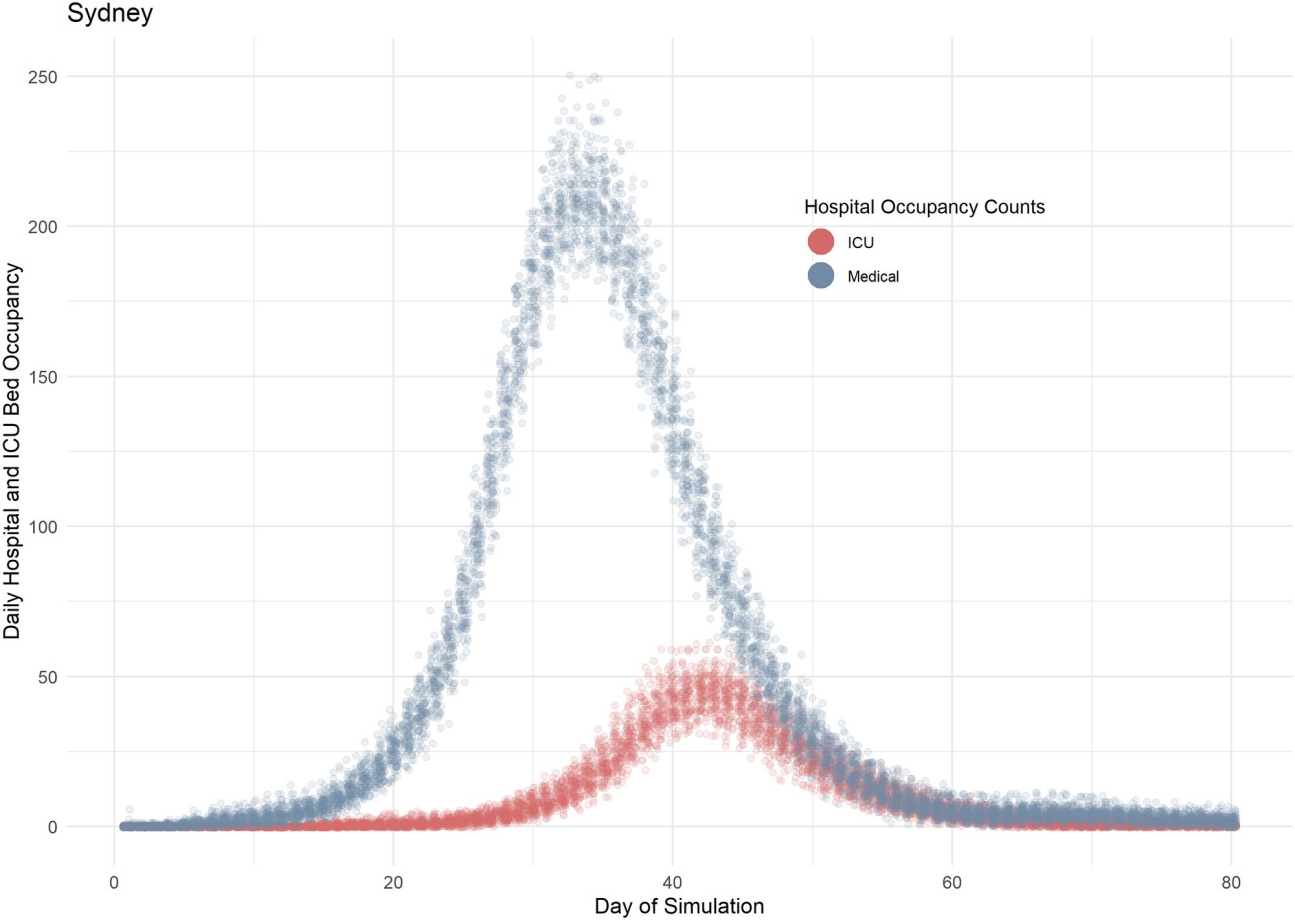
Simulated hospital occupancy in Sydney. The simulated prevalence of occupied medical and ICU beds is modelled as a function of the likelihood of requiring admission to hospital and subsequently requiring ICU care. The delayed peak in the ICU curve is demonstrative of the disease course, i.e. ICU requirements lag behind medical bed requirements. Each simulation is composed of 100 runs (each data point represents one day of simulation) which generates the spread of results seen here and the stochasticity that is inherent to ABMs.

**Fig 4:**
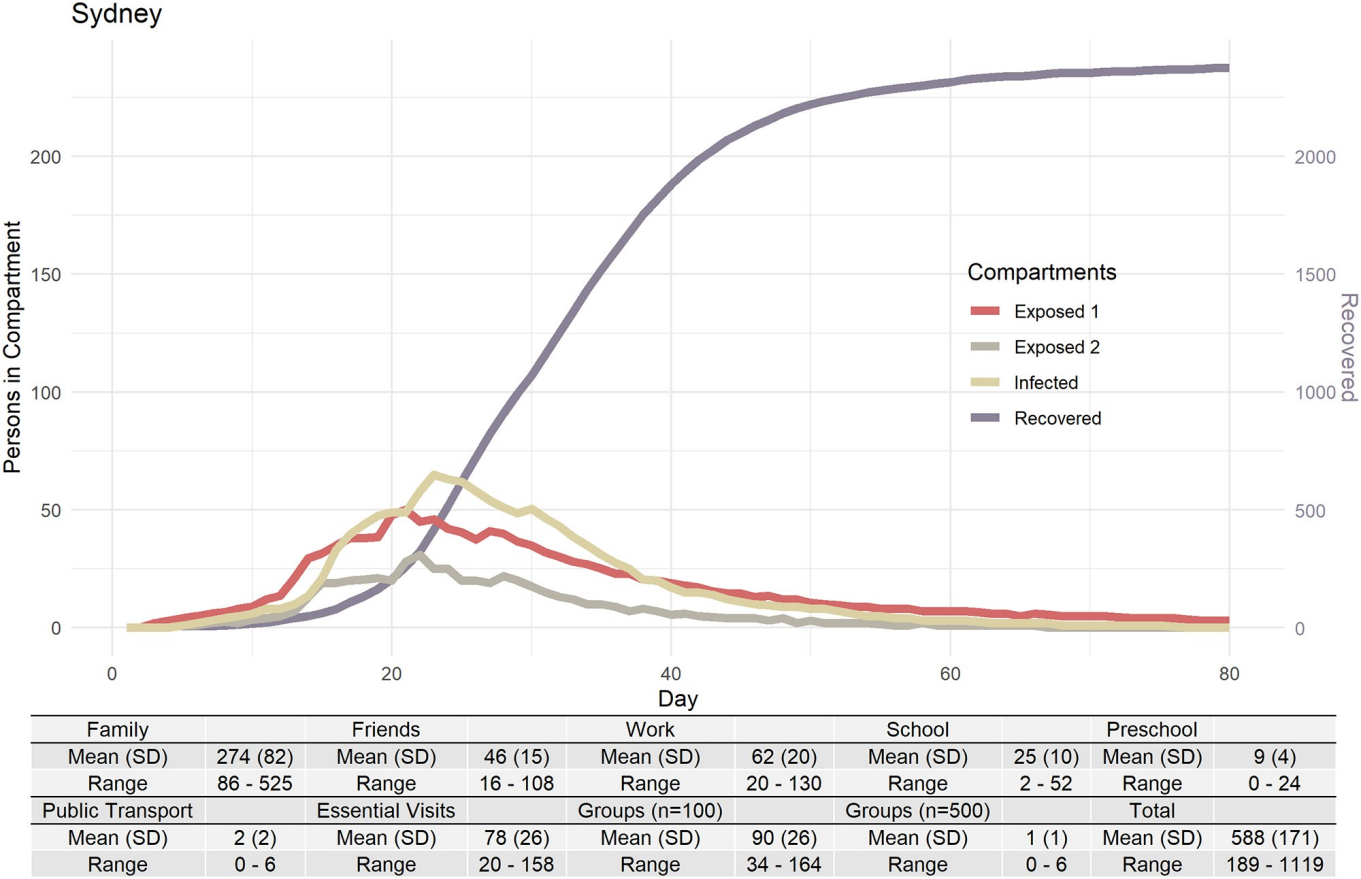
Four of the six SEIRD+M compartments shown with transmission counts within each of the containers. Container C_2_ (work and education) comprises three sub-containers (work, preschool and school).

Fig 3 shows plots of mean values (of 100 Sydney simulations) for hospitalisations and ICU admissions, showing the spread of results seen over 100 runs with the stochasticity inherent in DESSABNeT. Fig 4 shows SEIRD transmissions (susceptible compartment not shown), again demonstrating the stochasticity seen in simulation. Transmission in different containers is also shown in Fig 4, with most transmission occurring in families, workplaces and medium-sized gatherings.

**Fig 5:**
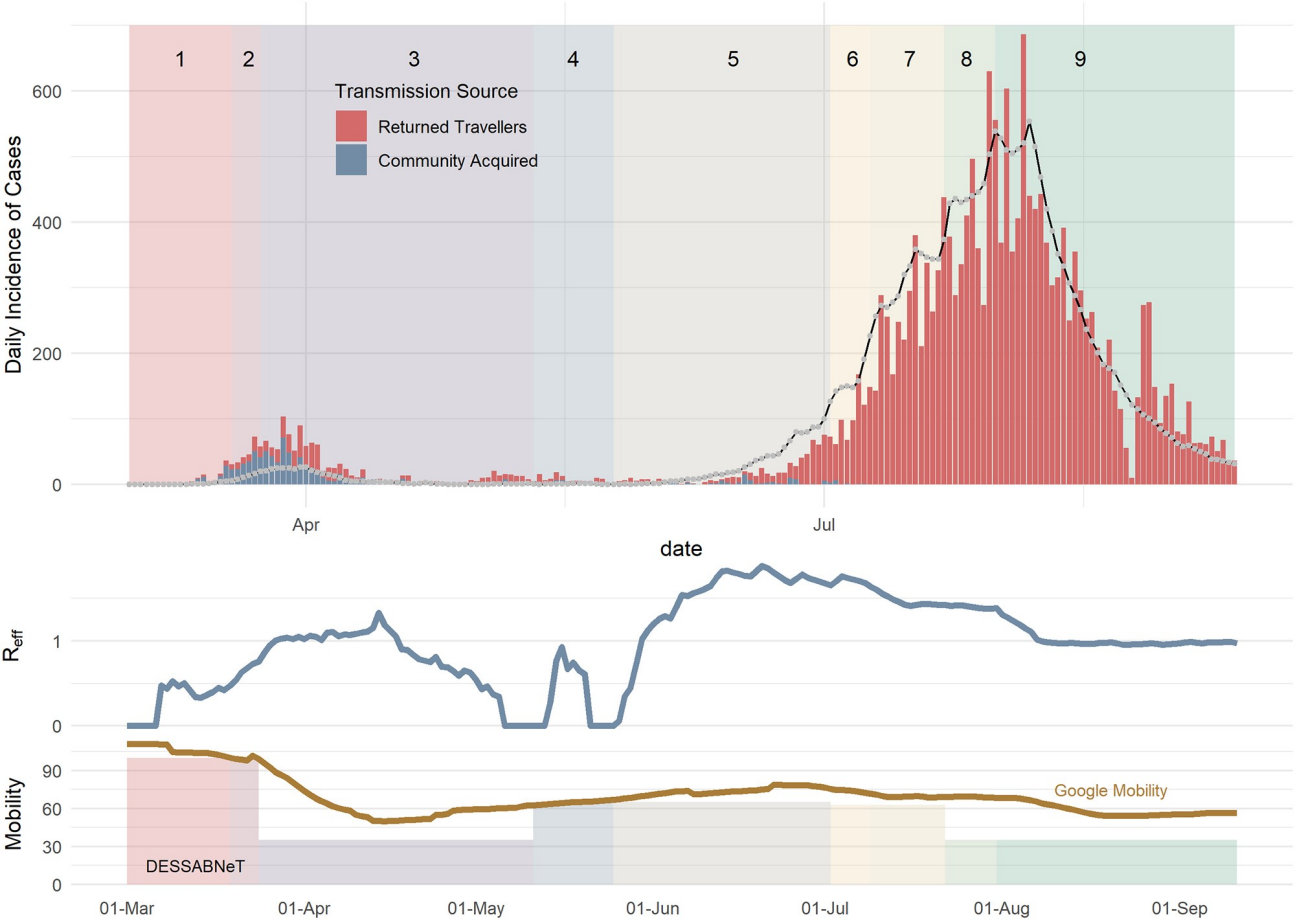
Melbourne daily incidence of COVID-19 cases (bars) vs simulated daily incidence of symptomatic cases (lines), with the simulated daily *R*_*eff*_ shown in the middle plot. The line in the top plot is the simulated total incidence. The rates of working from home used in our simulation is similar to observations in Google mobility (workplace) measures.

The Melbourne outbreak (population 4,967,730) was simulated for 195 days, comprising the first outbreak in March 2020, and the second wave of predominantly sustained community transmission from June 2020 through to 11 September 2020 (see Fig 5) [34]. The observed vs simulated estimates were drawn from the same distribution (p = 0.17). The median, 75^th^ and 95^th^ percentile of the maximum *R*_*eff*_ for each of the 100 simulations was 1.91, 2.17 and 3.26 respectively. Similarly, the median, 75^th^ and 95^th^ percentile of the maximum daily incidence in each of the 100 simulations was 578, 708 and 975, respectively.

**Fig 6:**
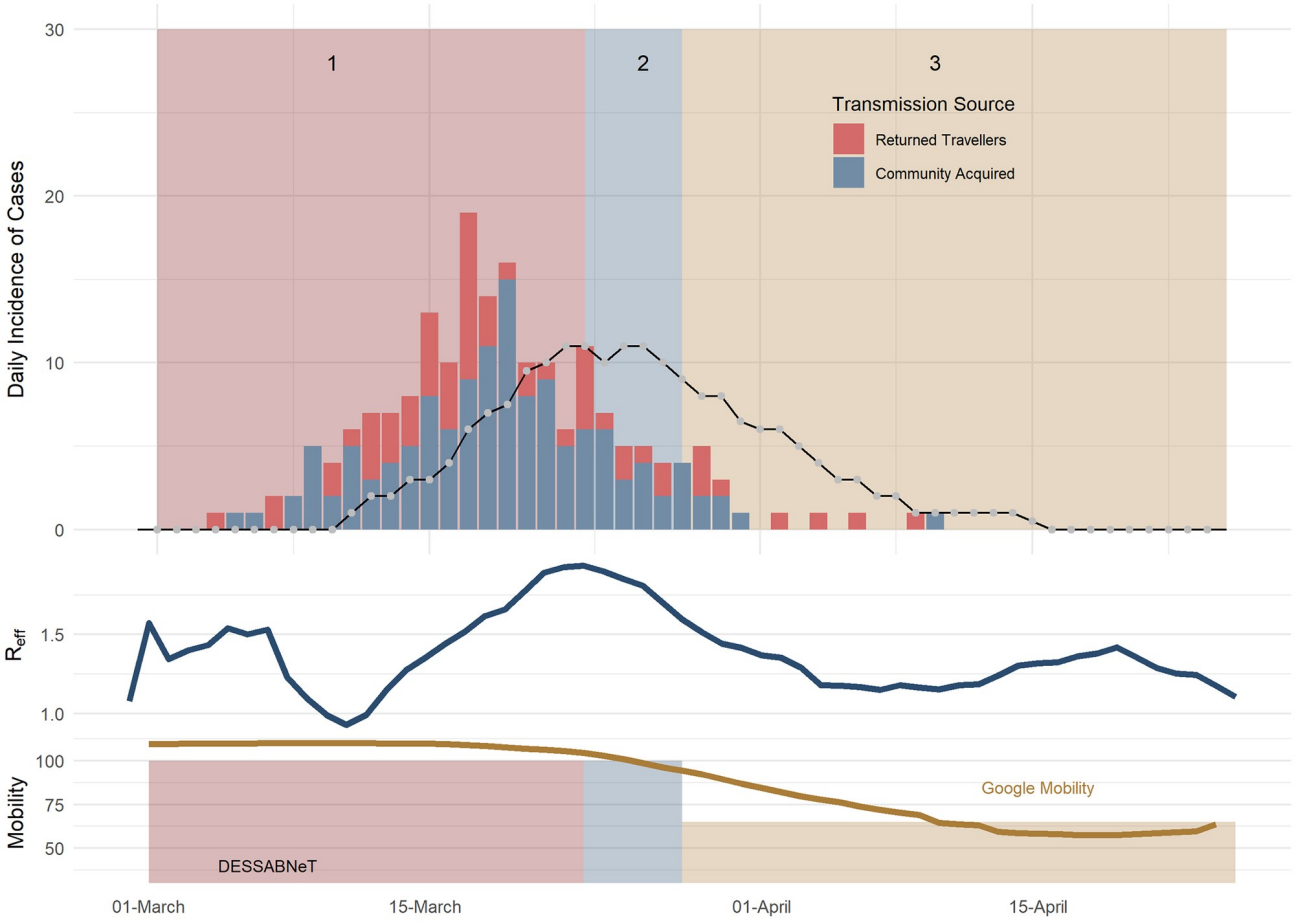
Gold Coast daily incidence of SARS-COV-2 cases (bars) vs simulated daily incidence of symptomatic cases (lines), with the simulated daily *R*_*eff*_ shown in the middle plot. The line in the top plot is the simulated total incidence. The rates of working from home used in our simulation is similar to measures performed by Google mobility (workplace) measures.

The Gold Coast outbreak with a population of 620,520 agents was simulated for 70 days (16 February– 25 April, 2020), with three social restriction phases. The fit was poorer with observed vs simulated estimates likely drawn from a different distribution statistically significant (p = 0.02), likely due to low case numbers) (see Fig 6). The median, 75^th^ and 95^th^ percentile of the maximum *R*_*eff*_ for each of the 100 simulations was 1.94, 1.96 and 1.98. Similarly, the median, 75^th^ and 95^th^ percentile of the maximum daily incidence in each of the 100 simulations was 16, 18.2 and 25, respectively.

For the Gold Coast simulation, the incidence of hospitalisations, ICU admissions and deaths were compared with real data. In Gold Coast, 100 simulations saw a median peak of 3 daily hospitalisations, compared to a peak of 7 real daily hospitalisations to Gold Coast hospitals and a simulated median peak of 1 daily ICU admission compared to 2 real ICU admissions to Gold Coast hospitals. Lastly, we saw median of 0 simulated deaths, while 0 actual deaths were recorded.

Table 5 presents the median of the maximum emergent R_eff_ values. Note that the emergent R_eff_ values lie within the bounds of the next generator operator R_0_ values for their respective cities, as seen in Tables 3–6. Melbourne saw the greatest median peak of simulated incidence, hospitalizations, ICU admissions and deaths per 100,000 population.

**Table 5:** Summary counts (and per 100,000 population estimate) of each simulation.

City	Emergent R_eff_	Incidence	Hospitalizations	ICU Admissions	Deaths
Sydney	1.87	94 (1.8)	25 (0.5)	7 (0.1)	2 (<0.1)
Melbourne	1.91	577.5 (11.65)	58 (1.15)	17 (0.3)	4 (0.1)
Gold Coast	1.94	16 (2.6)	3 (0.5)	1 (0.2)	0 (0)

Each simulation is composed of 100 simulations of 180 days. Each number represents the median value of the maximums or peak incidence of each simulation. Hospitalization, ICU admission and deaths are presented as daily incidences. The median of 100 peaks of Sydney’s R_eff_ was 1.87, indicating that the 50^th^ percentile of the peak R_eff_ for 100 simulations was 1.87. Pr(Death) = Pr(Death | ICU admission | Hospitalization).

**Table 6:** Social restrictions for the Gold Coast simulation.

Variable	Phase 1 (Normal Social Network)	Phase 2 (SR 1)	Phase 3 (SR 2)
Day Number	1 to 37	38 to 42	43 to 70
Dates	16 February—23 March, 2020	24 March—28 March, 2020	29 March—25 April, 2020
Melbourne Restrictions	Nil	Large gatherings cancelled. Pubs, hotels, clubs, restaurants and recreation facilities are closed.	26 March: Border closure, interstate travellers must self-quarantine for 14 days. 27 March: Queenslanders cannot have more than 10 people in their house at any one time. People are asked to stay at home where possible. 28 March: Overseas travellers must self-isolate for 14 days. Schools closed as of Monday 30 March, 2020
Restrictions in DESSABNeT	Nil*	↑ HLT, ↑ WFH, ↓ MGA, ↓ LGA, ↓ PTU, ↓ FKV	↑↑ HLT, ↑↑ WFH, 0% MGA, 0% LGA, ↓↓ PTU, ↓↓ FKV
Essential visits per week (N)	3	3	3
% large-group activity in leisure time	11.4	0	0
% medium-group leisure activity in leisure time	40.4	23	0
% solitary home activity in leisure time	48.2	77	100
% of agents working from home	30.2	47.1	64
% of agents using public transport	4.1	1.8	1.1
Friend and Kin Contacts per Week (N)	5	3	3
School attendance	Schools Open	Schools Open	Schools Closed 30 March
Median Phase R_0_ (95% CI)	1.459 (0.234, 6.417)	1.049 (0.149, 5.639)	0.754 (0.093, 4.953)

*Maximum large group exposure set at 500 agents from beginning of simulation.

HLT = Home Leisure Time, WFH = Work From Home, MGA = Medium Group Activity, LGA = Large Group Activity, PTU = Public Transport Use, FKV = Friend and Kin Visits.

Demonstrates the changing social network parameters as different restrictions are placed or lifted. Median phase R_0_ using the next generation operator represents the median basic reproduction number for the contact network with or without social restrictions in place. The 95% CI uses the 2.5^th^ and 97.5^th^ percentile of contact matrix values to provide a range of next generation operator R_0_ values.

## Discussion

We demonstrate a novel ABM designed to simulate the spread of communicable diseases in different population centres and have modelled the imposition and lifting of various social restrictions, demonstrating the effect of changing the connectivity of a social network (through social restrictions) on emergent outcomes such as *R*_*eff*_, hospitalization, ICU admissions and deaths. The emergent *R*_*eff*_ occurs within the bounds of a social network specific next generation operator R_0_. Calculated *R*_*eff*_ peaks compared well with recent *R*_*eff*_ estimates for March 2020 outbreaks in Australia [35].

The strengths of our system include the flexibility and granularity to accurately model a wide range of social restrictions and their easing, fluidly transitioning between different types and combinations of social restrictions within the same simulation. Though the fit was poorer, we were able to provide reasonable estimates in low incidence settings such as the Gold Coast.

Secondly, the DESSABNeT platform has a large number of modifiable parameters. The ability to alter network connectivity as well as disease dynamics (such as TP) allows modelling of different social restrictions, but also different (and even multiple) strains of SARS-CoV-2. Different vaccination strategies can also be simulated, modelling different efficacy and effectiveness parameters.

Thirdly, the software is able to run using widely available, modest computing resources, in contrast to other ABMs [1].

### Limitations

We acknowledge over-simplifications and assumptions in our model, many of which are common to ABMs. As a simplification, this system is an entirely closed system, with no births or deaths. In addition, we do not model geospatial networks.

ABMs use discrete time periods for modelling agent interactions, during which agents are usually devoted to one activity, so only one type of interaction can occur (attending school or work, being home or using public transport). While other systems use only two 12 hour time periods [1], DESSABNeT uses three time periods (approximately 8 hours each). Despite the detailed weekly agent schedules and entry into different containers creating a complex network of agent interactions, we acknowledge that persons within a container will mix homogenously.

Simplifications have also been introduced into disease dynamics: Infectivity during the infective period is of a uniform distribution (i.e. no crescendo-decrescendo infectivity as would be expected in real life). No agent can die without being hospitalized and then admitted to ICU.

We note that while our percentage of admissions from symptomatic infections and ICU admissions as a percentage of medical admissions were derived from Moss et al. and Zhou et al. [28,29], these have also been estimated for Australian data by others such as Price [35]. Price et al have hospitalisation rates from confirmed cases ranging from 10.81%–14.75% (age 0–18), 5.04%–15.29% (age 19–69), up to 38.15% (age 80+ years), and ICU admission rates from hospital cases of 0%-8.33% (age 0–18), 1.39%-20.80% (age 19–69), 22.87% (age 70–79) and 12.11% (age 80+), only some of which are comparable to the rates used here (Table 2). Price et al. have noted limitations in making their estimates, including that 31% and 58% of examined cases in their analysis had no information recorded under hospitalisation or ICU status, respectively. We recommend that future versions of DESSABNeT use multiple age bands and that variables used for medical outcomes be adjusted as further literature comes to light.

### Simulation and prediction

DESSABNeT simulations provide a range of outputs and outcomes. By altering input parameters, the effect of contact tracing, super-spreader events and social restrictions on the R_eff_ can be estimated. This platform allows modelling of different social restrictions, but also different (and even multiple) strains of SARS-CoV-2.

Modelling of COVID-19 vaccination is a priority—e.g. assessing individual protection in any given city or region as determined by number of individuals vaccinated as compared with putative herd immunity thresholds, based on vaccine properties of efficacy and effectiveness. We propose using DESSABNeT to address complex questions such as the optimal non-pharmaceutical interventions in the presence of differing vaccination strategies and SARS-CoV-2 strains, with impact on medical sequelae such as ICU admissions and deaths.

The effects of a communicable disease and non-pharmaceutical interventions on vulnerable or unique population groups (e.g. nursing homes, elderly or mining sites) can also be simulated. Of specific interest is simulating disease spread in rural communities which are geographically spread out but still connected by social and occupational networks. In addition, the system provides key outputs that can be used for strategic workforce purposes such as health resource utilisation requirements and contact tracing workforce requirements.

## Supporting information

S1 Table (XLSX)

S2 Table (XLSX)

S3 Table (XLSX)

S4 Table (XLSX)
